# Linoleic Acid Upregulates Microrna-494 to Induce Quiescence in Colorectal Cancer

**DOI:** 10.3390/ijms23010225

**Published:** 2021-12-25

**Authors:** Ruiko Ogata, Shiori Mori, Shingo Kishi, Rika Sasaki, Naoya Iwata, Hitoshi Ohmori, Takamitsu Sasaki, Yukiko Nishiguchi, Chie Nakashima, Kei Goto, Isao Kawahara, Rina Fujiwara-Tani, Hiroki Kuniyasu

**Affiliations:** Department of Molecular Pathology, Nara Medical University School of Medicine, Kashihara 634-8521, Japan; pkuma.og824@gmail.com (R.O.); m.0310.s.h5@gmail.com (S.M.); nmu6429@yahoo.co.jp (S.K.); rika0st1113v726296v@icloud.com (R.S.); niwata@naramed-u.ac.jp (N.I.); brahmus73@hotmail.com (H.O.); takamitu@fc4.so-net.ne.jp (T.S.); yukko10219102@yahoo.co.jp (Y.N.); c-nakashima@naramed-u.ac.jp (C.N.); ilgfgtk@gmail.com (K.G.); isao_kawahara@a011.broada.jp (I.K.); rina_fuji@naramed-u.ac.jp (R.F.-T.)

**Keywords:** linoleic acid, quiescence, dormancy, energy metabolism, delayed metastasis

## Abstract

Cancer dormancy is a state characterized by the quiescence of disseminated cancer cells, and tumor recurrence occurs when such cells re-proliferate after a long incubation period. These cancer cells tend to be treatment resistant and one of the barriers to successful therapeutic intervention. We have previously reported that long-term treatment of cancer cells with linoleic acid (LA) induces a dormancy-like phenotype. However, the mechanism underpinning this effect has not yet been clarified. Here, we investigate the mechanism of LA-induced quiescence in cancer cells. We first confirmed that long-term treatment of the mouse colorectal cancer cell line CT26 with LA induced quiescence. When these cells were inoculated subcutaneously into a syngeneic mouse and fed with an LA diet, the inoculated cancer cells maintained the quiescent state and exhibited markers of dormancy. LA-treated CT26 cells showed reduced oxidative phosphorylation, glycolysis, and energy production as well as reduced expression of the regulatory factors *Pgc1α* and *MycC*. MicroRNA expression profiling revealed that LA induced an upregulation in miR-494. The expression of *Pgc1α* and *MycC* were both induced by an miR-494 mimic, and the LA-induced decrease in gene expression was abrogated by an miR-494 inhibitor. The expression of miR-494 was enhanced by the mitochondrial oxidative stress produced by LA. In a syngeneic mouse subcutaneous tumor model, growth suppression by an LA diet and growth delay by LA pretreatment + LA diet were found to have similar effects as administration of an miR-494 mimic. In contrast, the effects of LA were abrogated by an miR-494 inhibitor. Analysis of human colorectal cancer tissue revealed that miR-494 was present at low levels in non-metastatic cases and cases with simultaneous liver metastases but was expressed at high levels in cases with delayed liver metastases, which also exhibited reduced expression of *PGC1α* and *MYCC*. These results suggest that miR-494 is involved in cancer dormancy induced by high levels of LA intake and that this microRNA may be valuable in targeting dormant cancer cells.

## 1. Introduction

The incidence of colorectal cancer (CRC), a leading cause of cancer-related deaths worldwide, has increased in recent years [1]. In the United States, nearly 150,000 cases of colorectal cancer and more than 50,000 deaths were reported in 2020 [1]. Many causes of colorectal cancer are related to lifestyle; Westernization of eating habits, obesity, and a lack of exercise are known risk factors for this cancer. Indeed, colon carcinogenesis is closely related to diet. Inflammation caused by fatty acid metabolism due to the fact of lipid overdose, alcohol abuse, and overconsumption of processed meat is known to increase the risk of tumor progression [2]. Furthermore, n-6 polyunsaturated fatty acids, such as linoleic acid (LA), are known to be involved in colorectal carcinogenesis due to the fact of free radicals generated by cyclooxygenase during prostaglandin E2 production [3,4].

In contrast, when LA is metabolized by 15-lipoxygenase-1, it activates peroxisome proliferator-activated receptor-γ, inhibits cancer cell proliferation and invasion, and induces apoptosis, thereby exerting a tumor suppressive effect [5,6]. Furthermore, long-term treatment with LA has been shown to induce quiescence and dormancy in cancer cells, although it can also result in a treatment-refractory phenotype [7]. Arachidonic acid and epoxyeicosatrienoic acid are also associated with the indication of cancer dormancy [8,9].

Cancer dormancy has been associated with late recurrence and metastasis in cancer patients, and incubation periods can range from years to decades [10]. In colorectal cancer, cases of recurrence have been reported several years after tumor resection at the primary site [11,12]. Dormancy involves quiescence of the tumor at the primary site or the metastatic lesion [13]. Cancer dormancy refers both to tumor mass dormancy (in which cell proliferation and cell death are balanced in the tumor) and to cell dormancy (in which cancer cells are in a quiescent state) [14]. Cellular dormancy can be induced by the extracellular matrix environment, a metastatic niche, a hypoxic microenvironment, and endoplasmic reticulum stress [13]. Tumor dormancy is one of the mechanisms of resistance to various cancer therapies, and the cells exhibit a stem cell-like phenotype that includes chemoresistance, high metastatic potential, and suppression of antitumor immunity [14,15,16,17]. At the same time, cell cycle progression is reduced in these cells, which is a defining feature of quiescent cancer stem cells [18]. However, the mechanism by which dormancy occurs in cancer cells is not entirely clear. Elucidating the mechanisms of cancer dormancy may help to improve treatment responsiveness by stimulating cancer cells to break dormancy after escape from treatment or the host immune system. Conversely, the development of tools to induce continued dormancy may also contribute to cancer control to avoid clinical recurrence.

microRNA (miRNA, miR)-494 is known to be overexpressed in tumors and is involved in angiogenesis by transferring to vascular endothelial cells via exosomes [19,20]. In contrast, in cancer cells, miR-494 has been reported to target *MYC* and *PGC-1α* [21,22,23,24,25]. MYC and PGC-1α are key regulatory proteins for glycolysis and oxidative phosphorylation, respectively, suggesting that miR-494 plays an important role in energy metabolism in cancer cells.

In our previous study, we demonstrated that in vitro treatment of cancer cells with LA induced quiescence [7]. In the present study, we used this system to investigate the mechanisms underpinning LA-induced quiescence.

## 2. Results

### 2.1. LA Induces Quiescence in CT26 Cells

**Figure 1:**
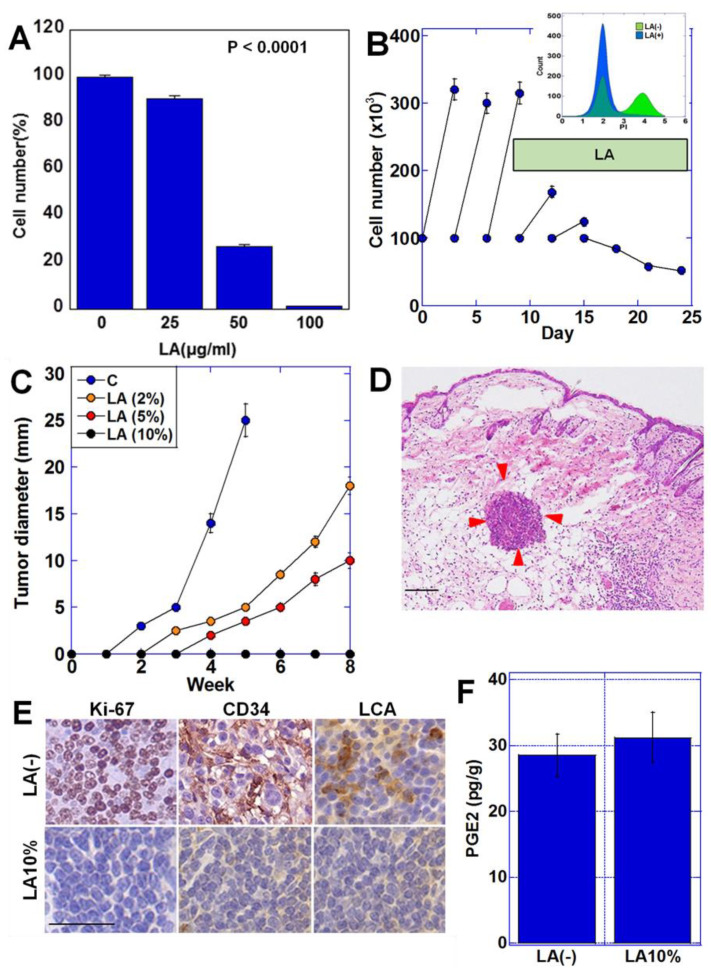
LA induced dormancy in CT26 cells. (**A**) Effect of short-term LA treatment on cell proliferation. (**B**) Effect of continuous LA treatment up to 25 days. (Insert) Cell cycle analysis of CT26 cells with or without continuous LA treatment. (**C**) Effect of LA on the growth of CT26-derived subcutaneous tumors in mice. Mice were fed a CE-2 standard diet containing LA (C, standard diet; 2%, 5%, and 10%, standard diet with LA (% *w*/*w*)). (**D**) Subcutaneous CT26 cell aggregation (red arrow). Hematoxylin and eosin staining. (**E**) Immunohistochemical analysis of proliferation (Ki-67), angiogenesis (CD34), and immune cell infiltration (LCA) of CT26 tumors in 10% LA-fed or standard diet-fed mice. (**F**) PGE2 concentration in CT26 tumors in 10% LA-fed or standard diet-fed mice. Scale bar, 100 μm. Error bar, standard deviation based on three independent trials. LA, linoleic acid; HPF, high-power field of view; LCA, lymphocyte common antigen; PGE2, prostaglandin E2.

We have shown in previous studies that long-term treatment with LA induces a quiescent state in cancer cells [7]. In the present study, in order to re-examine this result, we first examined the effect of LA on proliferation using CT26 colon cancer cells. Our results showed that cell proliferation was suppressed in a concentration-dependent manner with an IC_50_ of 35 μg/mL (Figure 1A). When CT26 cells were treated with the IC_50_ concentration of LA for an extended period (up to 25 days), cell proliferation was gradually suppressed, leading to the attainment of a quiescent state (Figure 1B). In the cell cycle analysis, long-term LA treatment increased the G0/G1 population and decreased the S and G2/M population without an increase in the under G0/G1 population. To examine the effect in vivo, CT26 cells pretreated with LA at the IC_50_ concentration (35 μg/mL) were subcutaneously inoculated into syngeneic BALB/c mice (Figure 1C). Tumor growth was suppressed in a concentration-dependent manner when the inoculated mice were fed a diet containing LA. Notably, mice fed with a 10% LA diet did not form palpable tumors. However, tumor cell aggregation was observed in the tissues at the subcutaneous inoculation sites in these mice (Figure 1D), although we did not detect immune cell infiltration or tumor vascularity in the tumor cell aggregations (Figure 1E). The results from MIB1 immunostaining indicated that cancer cell proliferation was significantly suppressed in the 10% LA diet group: 64 ± 5/high-power fields of view (HPF) in control mice vs. 4 ± 0.3/HPF in 10% LA-treated mice (*p* < 0.001). We also detected intratumoral prostaglandin E2 concentration (Figure 1F). In the CT26 cells in mice fed with a 10% LA diet, the PGE2 concentration was not different from that in the standard diet-fed mice. These results suggest that LA inhibits proliferation in cancer cells.

### 2.2. LA Treatment Altered Intracellular Energy Metabolism

**Figure 2:**
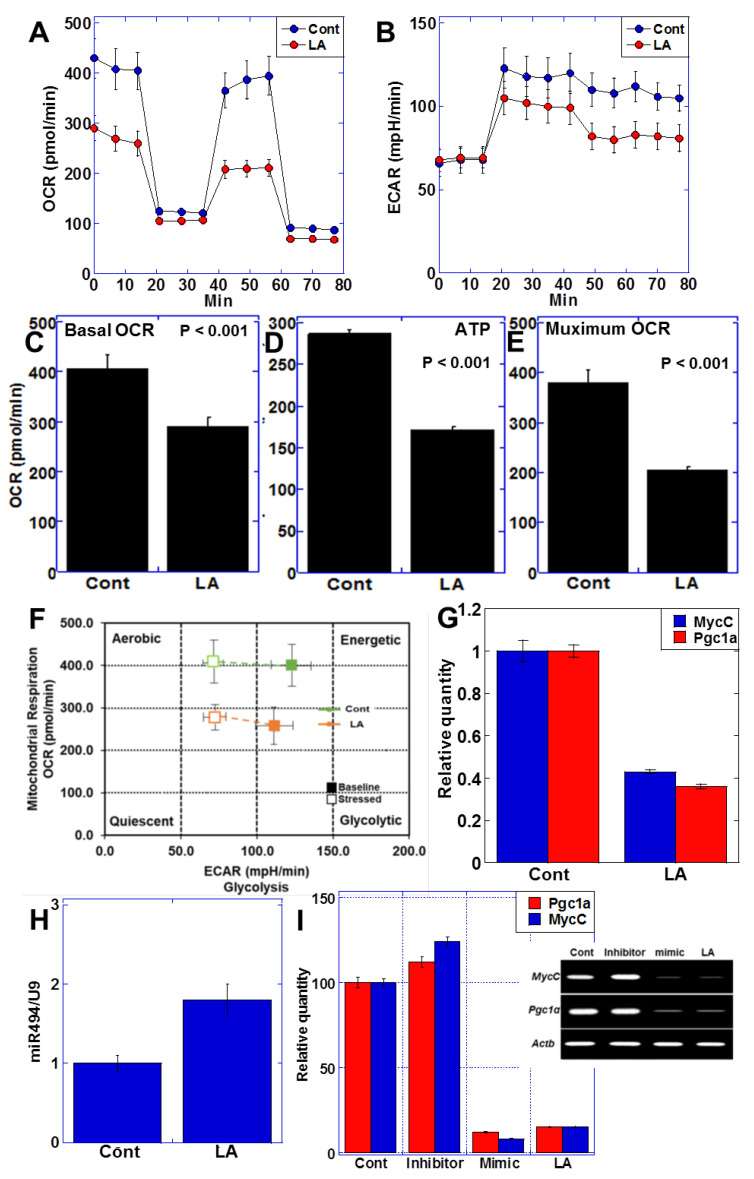
LA treatment lowered the energy metabolism in CT26 cells. (**A**) Effect of LA (35 μg/mL) on mitochondrial respiration. (**B**) Effect of LA (35 μg/mL) on glycolytic activity (determined by ECAR measurement). (**C**–**E**) Effect of LA on basal OCR, ATP production, and maximum OCR. (**F**) Effect of LA on cell energy phenotype profile. (**G**) Effect of LA on expression of *Pgc-1α* and *MycC*. (**H**) Effect of LA on expression of miR-494. (**I**) Effect of miR-494 inhibitor (inhibitor, co-treated with LA), miR-494 mimic (mimic), and LA on expression of *Pgc-1α* and *MycC*. The expression was examined by quantitative RT-PCR. (Right) Ethidium bromide image of RT-PCR. Error bars, standard deviation based on three independent trials. LA, linoleic acid; OCR, oxygen consumption rate; ECAR, extracellular acidification rate; ROS, reactive oxygen species; PGC, peroxisome proliferator-activated receptor gamma coactivator; ACTB, actin β.

We next examined the intracellular energy metabolism of CT26 cells treated with LA (35 μg/mL) (Figure 2A,B). LA treatment resulted in a decrease in both oxidative phosphorylation (oxygen consumption rate, OCR) and glycolysis (extracellular acidification rate, ECAR), and basal OCR, ATP production, and maximum OCR all decreased in comparison with the control (Figure 2C–E). Furthermore, matrix analysis of OCR and ECR suggested that LA treatment induced a quiescent cell state (Figure 2F).

Given these LA-induced alterations in the intracellular energy metabolism, we examined the expression of *MycC*, a gene that promotes glycolysis [26], and peroxisome proliferator-activated receptor γ coactivator 1α (*Pgc1α*), a gene that promotes oxidative phosphorylation [27]. Our analyses showed that LA treatment repressed the expression of both *MycC* and *Pgc1α* (Figure 2G). We also examined miRNA expression in LA-treated CT26 cells using microarray analysis (Table 1), which revealed that the expression of miR-494 increased by approximately two-fold in response to LA treatment (Figure 2H). As the miRNA database suggested that miR-494 targets *MycC* and *Pgc1α*, we investigated the effects of an miR-494 inhibitor and mimic on *MycC* and *Pgc1α* gene expression (Figure 2I). Treatment with miR-494 mimics suppressed the gene expression of *MycC* and *Pgc1α* in the same manner as LA treatment. In contrast, treatment with the LA and miR-494 inhibitor maintained the expression of both genes at the same level as that of the control, suggesting that repression of *MycC* and *Pgc1α* might be caused by LA-induced miR-494.

### 2.3. LA Induces Oxidative Stress

**Figure 3:**
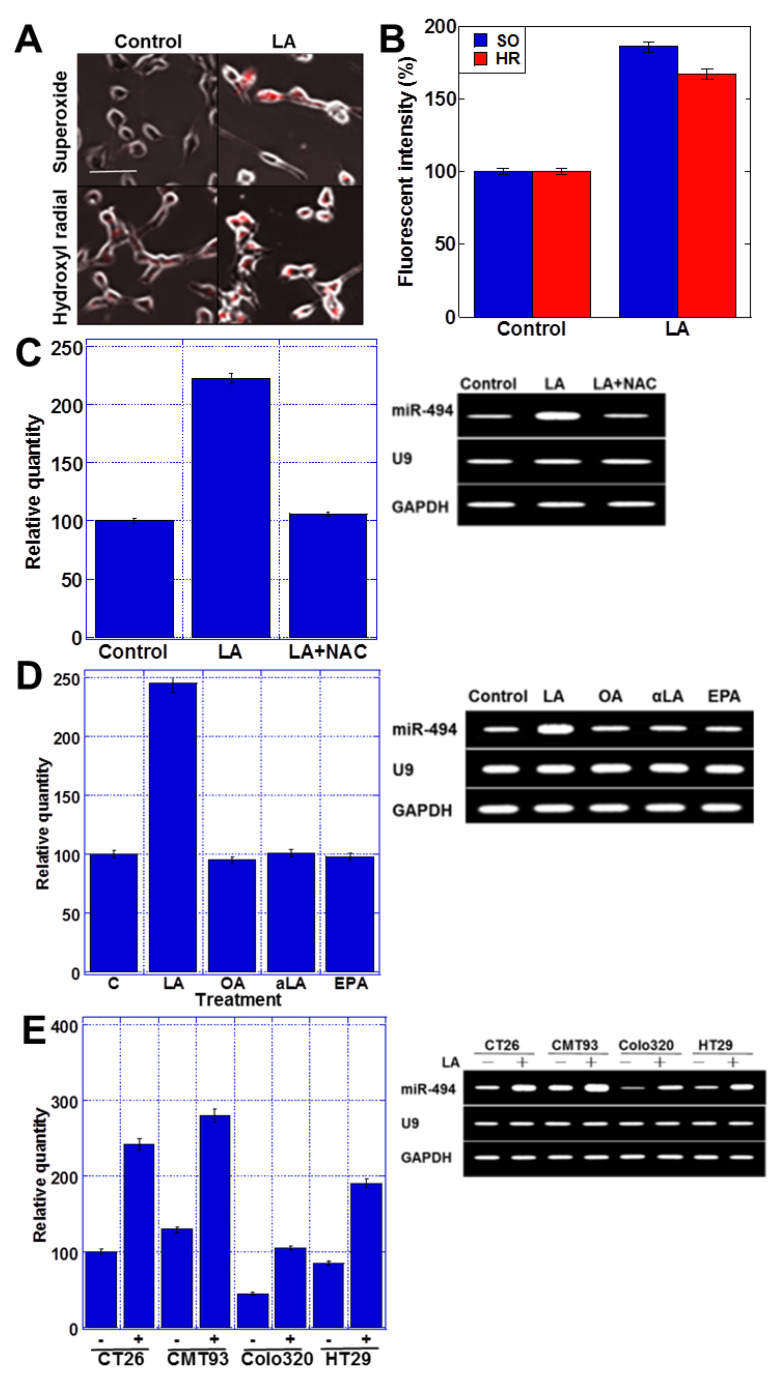
LA-induced mitochondrial ROS upregulated miR-494. (**A**) Mitochondrial superoxide and hydroxy radicals visualized by fluorescent dye probes. Scale bar, 50 μm. (**B**) Fluorescent intensities of mitochondrial superoxide and hydroxy radicals. (**C**) Expression of miR-494 in CT26 cells treated with LA and/or NAC examined by quantitative RT-PCR. (Right) Ethidium bromide image of RT-PCR. (**D**) Expression of miR-494 in CT26 cells treated with LA, oleic acid (OA, 35 μg/mL), α-linolenic acid (αLA, 35 μg/mL), and eicosapentaenoic acid (EPA, 40 μg/mL) for 48 h. (Right) Ethidium bromide image of RT-PCR. (**E**) Expression of miR-494 in CT26, CMT93, Colo320, and HT29 CRC cell lines was examined with or without LA treatment (35 μg/mL, 48 h). (Right) Ethidium bromide image of RT-PCR. Error bars, standard deviation from three independent trials. LA, linoleic acid; SO, superoxide; HR, hydroxy radical; NAC, N-acetyl-L-cysteine; GAPDH, glyceraldehyde-3-phosphate dehydrogenase.

The expression of many miRNAs is regulated by oxidative stresses [28], and we therefore next examined the effect of LA on mitochondrion-derived oxidative stress (Figure 3). We observed that the levels of mitochondrial superoxides and hydroxy radicals were increased by LA treatment (Figure 3A,B) and that the reduction of reactive oxygen species (ROS) by N-acetyl-l-cysteine (NAC) abrogated the LA-induced upregulation of miR-494 expression (Figure 3C). These data suggested that LA-induced ROS might upregulate miR-494. We examined the effect of other unsaturated long-chain fatty acids on miR-494 expression (Figure 3D). Differently from LA, oleic acid (OA), α-linolenic acid (αLA), and eicosapentaenoic acid (EPA) did not affect miR-494 expression. We also examined the miR-494 induction by LA in other CRC cell lines (Figure 3E). In all four CRC cell lines (i.e., CT26, CMT93, Colo320, and HT29), LA treatment increased miR-494 expression.

### 2.4. miR-494 Induced Quiescence in CT26 Cells in Syngeneic BALB/c Mice

**Figure 4:**
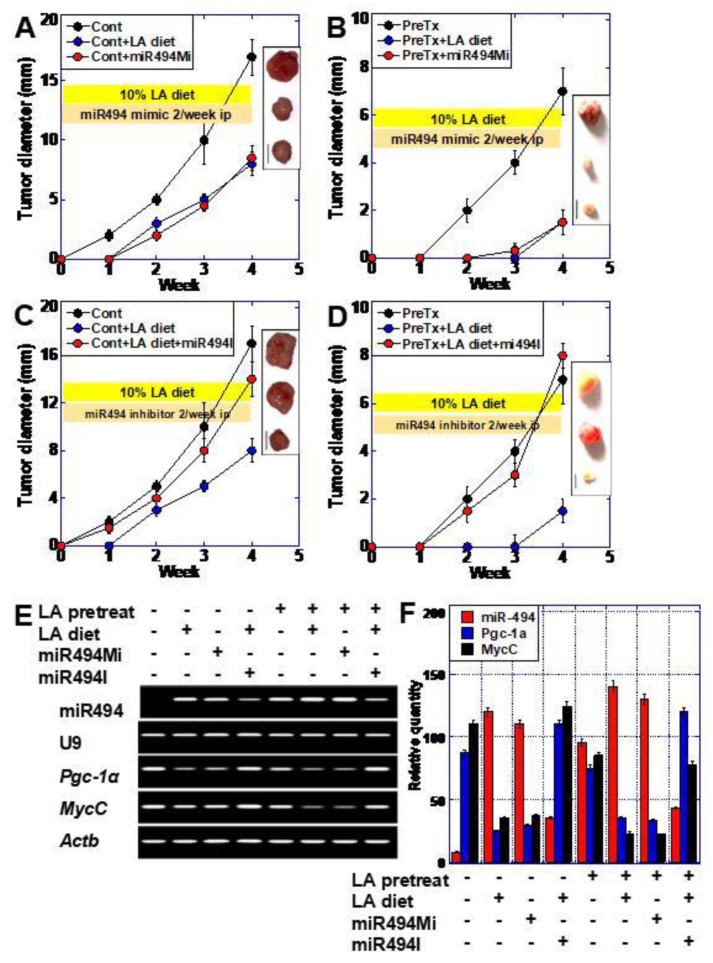
miR-494 mediated LA-induced growth impairment in a mouse model. (**A**,**C**) Effect of miR-494 mimic (miR-494Mi) or miR-494 inhibitor (miR-494I) on LA-induced growth inhibition. CT26 cells (1 × 10^7^) were subcutaneously inoculated into BALB/c mice, which were fed a control or 10% LA diet. (Insert) Example image of excised tumors. Cont, Cont+LA, Cont+Mi or I, from the top. (**B**,**D**) Effect of miR-494 mimic (miR-494Mi) or miR-494 inhibitor (miR-494I) on LA-induced growth delay. LA-pretreated (100 μg/mL for 48 h) CT26 cells (1 × 10^7^) were subcutaneously inoculated into BALB/c mice, which were fed a control or 10% LA diet. (Insert) Example images of excised tumors. PreTx, PreTX+Mi or I, PreTx+LA, from the top. (**E**) Expression of miR-494, *Pgc-1α,* and *MycC* in each experimental group at week 4. *U9* and *Actb* expression were analyzed as loading controls. miR-494 mimic and miR-494 inhibitor were administered at a dosage of 7 mg/kg body weight intraperitoneally twice per week. (**F**) Expression of miR-494, *Pgc-1α,* and *MycC* in each experimental group at week 4 examined by quantitative RT-PCR. Error bar, standard deviations of data from 5 mice. Scale bar, 5 mm. LA, linoleic acid; PGC, peroxisome proliferator-activated receptor gamma coactivator; ACTB, actin β.

Using a syngeneic mouse subcutaneous model, we examined the effect of the miR-494 mimics or the inhibitor on CT26 cancer cells. Tumor growth of CT26 cells was inhibited by a 10% LA diet, while administration of the miR-494 mimic inhibited tumor growth in a similar manner, including the repression of *MycC* and *Pgc1α* (Figure 4A,E). CT26 cells pretreated with LA (100 µg/mL for one week) demonstrated delayed growth in comparison with vehicle-pretreated cells. Treatment with LA or the miR-494 mimic both prolonged the periods of growth suppression in a similar manner (Figure 4B). The repression of *MycC* and *Pgc1α* was also more pronounced in these cells compared to cells lacking pretreatment. In contrast, the miR-494 inhibitor abrogated both LA-induced growth inhibition in cells without pretreatment (Figure 4C) and LA-induced growth delay in LA-pretreated cells (Figure D). It also increased the expression of *MycC* and *Pgc1α* (Figure 4E). These results indicate that an LA diet or miR-494 mimic inhibited tumor growth by suppressing the expression of *MycC* and *Pgc1α*. In contrast, miR-494 inhibitor recovered tumor growth by canceling the LA-induced suppression of MycC and Pgc1α expression.

### 2.5. miR-494 Expression Is Associated with Delayed Liver Metastasis in CRC Patients

**Figure 5:**
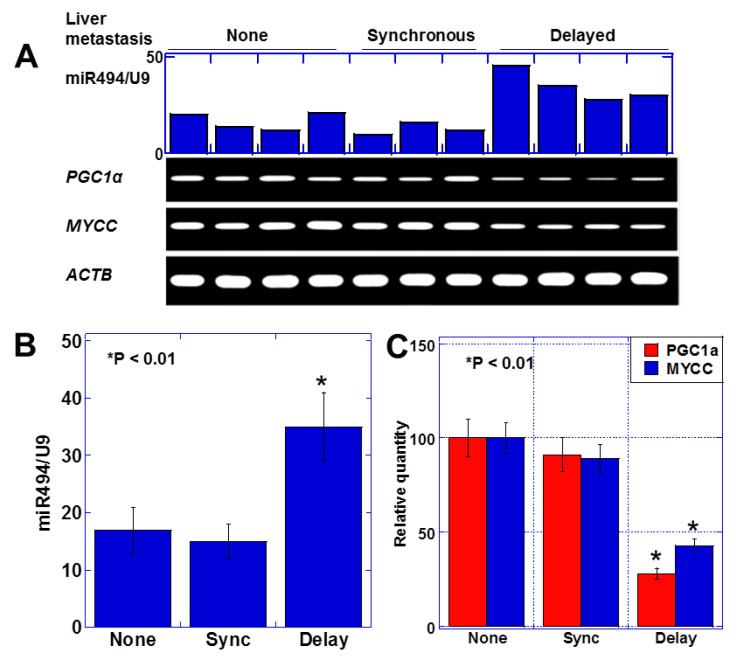
Expression of miR-494, *PGC-1α*, and *MYCC* in 11 human CRCs. (**A**) Expression of miR-494, *PGC-1α*, and *MYCC* in 11 human CRCs categorized as pT3/pN1. (**B**) Mean expression of miR-494. (**C**) *PGC-1α* and *MYCC* expression examined by quantitative RT-PCR. Error bar, standard deviation from three independent trials. None, no liver metastasis; synchronous (Sync), synchronous liver metastasis at primary operation; delayed (delay), liver metastasis delayed more than 2 years after primary operation. * *p* < 0.01.

Finally, we analyzed human CRC cases to investigate the clinical significance of miR-494 revealed in vitro and in vivo. We examined the expression of miR-494, *MYCC*, and *PGC1α* in primary tumors from 11 cases of CRCs (pT3/pN1) (Figure 5). In the four cases without liver metastasis and the three cases with synchronous liver metastasis, we observed low levels of miR-494 expression and high levels of *MYCC* and *PGC1α* expression. In contrast, all four cases with delayed liver metastasis showed miR-494 expression at high levels and low levels of *MYCC* and *PGC1α* expression (Figure 5A). In these cases, miR-494 levels demonstrated an inverse correlation with *MYCC* and *PGC1α* levels. The mean expression levels of miR-494 in the “delayed metastasis” cases were elevated to 196% of the “no metastasis” or “synchronous metastasis” cases (Figure 5B). In contrast, the levels of *MYCC* and *PGC1α* in the “delayed metastasis” cases were reduced to 47% and 41%, respectively, of the “no metastasis” or “synchronous metastasis” cases (Figure 5C). The observation of the increased expression of miR-494 and decreased expression of MYCC and PGC1α in cancer cells in the delayed metastasis cases suggests that the dormancy state may have been similar to that induced by LA or the miR-494 mimic in the mouse model.

These results showed that miR-494 expression was induced by LA, induced quiescence in CRC cells in vitro, induced dormancy in vivo, and correlated with delayed liver metastasis in clinical specimens.

## 3. Discussion

In this study, we showed that LA induced miR-494 expression via ROS and suppressed the expression of its target genes, *MYCC* and *PGC1α*, resulting in decreased energy production and quiescence. These in vitro results were compatible with our findings that cell quiescence is involved in tumor dormancy in animal models and delayed liver metastasis in human CRC.

In the present study, continuous treatment with LA induced dormancy in the mouse colon cancer cell line CT26, resulting in diminished proliferation, angiogenesis, and immune cell infiltration. Similar results have been reported in human colon and gastric cancer cell lines [7]. Dormancy is a quiescent state of the tumor at the primary site or in metastatic disseminated lesions [13]. The induction of quiescence has also been implicated in the increased stem cell phenotype, which is a feature of cancer cell dormancy [13,16]. In a clinical context, studies have reported that cancer dormancy is associated with the development of late recurrence and metastasis with latency periods of years to decades in cancer patients [10] as well as the acquisition of resistance to cancer treatment and escape from antitumor immunity [16], making it a distinct barrier to successful cancer treatment.

Our data revealed that LA-induced quiescence in CT26 cells is suppressing both oxidative phosphorylation and glycolytic energy metabolism. Furthermore, we observed a decrease in the expression of *Pgc1α* and *MycC*, which promote energy metabolism. These results suggest that LA suppresses energy metabolism in cancer cells and induces a quiescent metabolic state. These data are in line with previous reports that proteolysis of CMYC and decreased expression of CMYC [29,30] as well as the inhibition of SRC3-related PGC1α [31] are involved in the induction of quiescence. Myc regulates gene expression directly, as in glycolytic genes including lactate dehydrogenase A, or indirectly such as by repressing microRNA miR-23a/b to increase glutaminolysis via glutaminase expression [26]. PGC1α is a co-transcriptional regulation factor that induces mitochondrial biogenesis by activating different transcription factors, including nuclear respiratory factor 1 and nuclear respiratory factor 2, which activate mitochondrial transcription factor A [32]. As a result of enhanced mitochondrial biogenesis, oxidative phosphorylation is enhanced.

We also found that both LA-induced miR-494 expression and the administration of an miR-494 mimic led to the repression of *MycC* and *Pgc1α*. miR-494 is a key modulator of the G2-M phase of the cell cycle and suppresses cell growth [24,33]. The *MYCC* and *PGC1α* genes have been previously reported to be target genes of miR-494 [21,24,25], suggesting that LA suppresses the expression of both *MYCC* and *PGC1α* via the induction of miR-494. In particular, miR-494 is thought to have a strong effect on energy metabolism, because it simultaneously suppresses the expression of these key regulators of energy metabolism.

In this study, we found that miR-494 expression was induced by LA. The ability of fatty acids to regulate miRNA expression has been previously reported for oleic acid, which has been shown to promote the expression and processing of miR-7 and miR-16 [34]. In contrast, our data showed that LA-induced ROS upregulated miR-494 levels. Oxidative stress affects the expression levels of several miRNAs and, conversely, miRNAs regulate many genes involved in the oxidative stress response [35]. The increased expression of miR-494 by oxidative stress may be a negative feedback response to the generation of mitochondria-derived oxidative stress.

In this study, we examined 11 cases of human CRC cases, all at the same invasive depth and with lymph node metastasis. Importantly, we observed a marked increase in miR-494 and a decreased expression of *MYCC* and *PGC1α* in patients with liver metastases that occurred more than 2 years after resection of the primary lesion. In such cases, the cancer cells may have been latent in the dormancy state, and while we were unable to determine the patients’ intake of LA, the association between miR-494, MYCC, and PGC1α was confirmed. The involvement of miR-494 in the induction of dormancy is worthy of further analysis using a larger number of cases, especially in the context of breast cancer, in which dormancy is frequently experienced [36]. For examination of serum LA concentration, a prospective examination of colorectal cancer patients is needed in the future.

In this study, we suggest that LA induces quiescence by promoting miR-494 expression, resulting in dormancy of cancer cells. LA is thought to promote carcinogenesis of CRC, and our findings provide a new link between LA and this cancer. Our present findings suggest that miRNA expression may be affected by fatty acid intake in cancer patients, which may be a cause for concern. Breaking the dormancy of cancer is expected to become a new therapeutic target for cancer treatment in the future.

## 4. Materials and Methods

### 4.1. Cells

The mouse colon cancer CT26 cell line was provided by Isaiah J. Fidler (MD Anderson Cancer Center, Texas University, Houston, TX, USA). The CMT93 mouse colon cancer cell line was purchased from DS Pharma Inc., (Osaka, Japan). Colo320 and the HT29 human colon cancer cell lines were purchased from Dainihon Pharmaceutical Co. (Tokyo, Japan). The cells were cultured in Dulbecco’s modified Eagle’s medium (Wako Pure Chemical, Osaka, Japan) supplemented with 10% fetal bovine serum (Sigma Chemical Co., St. Louis, MO, USA) at 37 °C in a 5% CO_2_ atmosphere. LA, OA, αLA, and EPA (Wako) were dissolved in 100% ethanol (20 µg/mL), and the same quantity of 100% ethanol was used for control treatments.

### 4.2. Cell Growth

Cells (1 × 10^5^) cultured in six 3.5 cm diameter dishes were treated with LA (35 µg/mL). Cell viability was analyzed using the CellTiter AQueous One Solution Cell Proliferation Assay kit (Promega Corporation, Madison, WI, USA). Following treatment with LA, MTS (3-(4,5-dimethylthiazol-2-yl)-5-(3-carboxymethoxyphenyl)-2-(4-sulfophenyl)-2H-tetrazolium) solution was added to each well for 1 h at 37 °C in 5% CO_2_. Thereafter, absorbance at 490 nm was recorded using a microplate reader. Cells (1 × 10^5^) were also cultured with weekly re-seeding to examine the effects of long-term treatment with LA. The number of cells was counted using an autocytometer (Sysmecs, Kobe, Japan), and the experiment was repeated three times.

### 4.3. Cell Cycle Analysis

For analysis of the cell cycle distribution, the DNA content of individual cells was measured by flow cytometry. Core DNA content was measured using a linear amplification in the FL-2 channel of a FACScan flow cytometer (BD Biosciences, Franklin Lakes, NJ, USA).

### 4.4. Animal Model

Four-week-old male BALB/c mice were purchased from SLC Japan, Inc. (Shizuoka, Japan). The animals were maintained in a pathogen-free animal facility at 23 °C and 50% humidity, under a 12 h light/dark cycle. The animal study was conducted in accordance with the institutional guidelines approved by the Committee for Animal Experimentation of Nara Medical University, Kashihara, Japan, following current regulations and the standards of the Japanese Ministry of Health, Labor, and Welfare (approval no. 11528). The mice were used for approved experiments according to the institutional guidelines when they were 5 weeks old. To assess the effect of LA on the tumorigenicity of CT26 cells, the cells were pretreated with LA (100 µg/mL) for 5 weeks, with medium exchange and addition of LA every 7 days. Thereafter, 1 × 10^7^ cells were injected subcutaneously into the back of each mouse in each group. Tumor growth at the inoculation site was examined every 3 days after inoculation. After inoculation, the mice were fed either a control diet (CE-2, CLEA Japan, Inc., Tokyo, Japan) or an LA diet (a CE-2 diet containing 2%, 5%, or 10% *w*/*w* of LA).

### 4.5. Immunohistochemistry

Sections (4 µm thick) were immunostained with antibodies against the Ki-67 clone MIB1, lymphocyte common antigen (LCA), CD34 (DAKO Corp., Carpinteria, CA, USA), or single-stranded DNA (ssDNA, Enzo Life Science, Plymouth Meeting, PA, USA). Immunohistochemical staining was performed using the immunoperoxidase technique following antigen retrieval by microwave treatment (500 W, 5 min, 3 times) in citrate buffer (DAKO). After blocking with 3% H_2_O_2_–methanol for 10 min, the specimens were incubated with a primary antibody (0.5 μg/mL) for 2 h at room temperature. The specimens were briefly washed with PBS and incubated with a secondary antibody conjugated with peroxidase (0.5 μg/mL, Medical & Biological Laboratories Co., Ltd., Nagoya, Japan) for 1 h at room temperature. The specimens were then washed with PBS and treated with a stable diaminobenzidine solution (DAKO) for color development, followed by counterstaining with Mayer’s hematoxylin solution (Sigma). The number of cells with positive staining for Ki-67 (proliferation marker), CD34 (vessel), and LCA (immune cells) were counted, and the mean value was calculated from the microscopic observation of 30 high-power fields of view.

### 4.6. Enzyme-Linked Immunosorbent Assay (ELISA)

ELISA kits were used for measuring protein levels of PGE2 (Cayman Chemical Co., Ann Arbor, MI, USA). The assay was performed according to the manufacturer’s instructions, and whole cell lysates were used for the measurements.

### 4.7. Reverse Transcription-Polymerase Chain Reaction (RT-PCR)

To assess murine mRNA expression, RT-PCR was performed with 2 µg of total RNA extracted using TRI Reagent (Molecular Research Center, Inc., Cincinnati, OH, USA) according to the manufacturer’s protocol. cDNA was synthesized using a High Capacity cDNA Reverse Transcription Kit (Applied Biosystems, Carlsbad, CA, USA). The primer sets are listed in Table 2 and were synthesized by Sigma Genosys (Ishikari, Japan). PCR products were electrophoresed on a 2% agarose gel and stained with ethidium bromide. *GAPDH* mRNA was also amplified for use as an internal control.

### 4.8. Detection of miRNAs

A mirVana™ miRNA isolation kit was used to extract miRNAs according to the manufacturer’s protocol (Thermo Fisher Scientific, Waltham, MA, USA). For the quantification of miRNA expression levels, RT-PCR reactions were performed using the TaqMan miRNA reverse transcription kit (Applied Biosystems) and Pri-miRNA Assay kit (Hs04225959_pri, Applied Biosystems) according to the manufacturer’s protocols. 

### 4.9. Seahorse Assay (Mitochondrial Respiration)

To assess the metabolic activity in the CT26 cells, the OCR was measured using a Seahorse XFp Extracellular Flux Analyzer with Seahorse XFp FluxPaks (Agilent Technologies Inc., Santa Clara, CA, USA). The CT26 cells were cultured in regular medium for 72 h before plating the cells for the Seahorse assay in the recommended medium. For these experiments, we measured the OCR of 1 × 10^4^ viable CT26 cells per well.

Seahorse assays were carried out as follows: OCR (pmol/min) was measured before (basal OCR) and after successive injections of 3 µmol/L oligomycin (ATP synthase inhibitor), 2 µmol/L FCCP (carbonyl cyanide-p-trifluoromethoxy phenylhydrazone, an uncoupling protonophore), 0.5 µmol/L rotenone (Complex I inhibitor), and 0.5 µmol/L antimycin A (Complex III inhibitor). From the resulting data, we determined the OCR associated with respiratory ATP synthesis (oligomycin sensitive), the maximum OCR in FCCP-uncoupled mitochondria, the rotenone-sensitive OCR attributable to uncoupled Complex I activity, the antimycin-sensitive Complex II/III activity, and the OCR by mitochondrial functions other than ATP synthesis including the OCRs for mitochondrial membrane potential-driven (proton leak), non-respiratory oxygen consumption, and the respiratory “spare capacity” (excess capacity of the respiratory electron transport chain that is not being used in basal respiration).

### 4.10. Seahorse Assay (Glycolytic Respiration)

The ECAR of CT26 cells was measured using a modified glycolytic stress test in a Seahorse XF^e^24 Extracellular Flux Analyzer with Seahorse XFp FluxPaks (Agilent). CT26 cells were cultured in a growth medium in 6-well plates prior to the Seahorse experiments. CT26 cells (1 × 10^4^ cells/well) were plated in XF base medium (Agilent) containing 200 mmol/L L-glutamine and 5 mmol/L HEPES as recommended by the manufacturer of the glycolytic assays. The sensor cartridge apparatus was rehydrated 1 d in advance by adding 1 mL XF Calibrant to each well and incubating at 37 °C until needed. The injection ports of the sensor cartridge apparatus were loaded with the following drugs in chronological order of four injections to meet the indicated final concentrations in the wells: 10 mmol/L glucose, 1 µmol/L oligomycin, 1 µmol/L rotenone plus 5 µmol/L antimycin A (combined injection), and 50 mmol/L 2-deoxyglucose. Treatment with the rotenone/antimycin combination allowed for the assessment of the impact of electron transport on ECAR by respiratory acidification coupled with the passage of some glycolytic pyruvate through the tricarboxylic acid cycle to oxidative phosphorylation.

### 4.11. Mitochondrial ROS

Mitochondrial ROS was measured using a superoxide-sensitive dye (MitoROS 580, AAT Bioquest Inc., Sunnyvale, CA, USA), which selectively targets mitochondria. Briefly, washed CT26 cells (5 × 10^4^) were loaded with 1× MitoROS 580 dye in the dark for 30 min at 37 °C and then analyzed using a fluorescence microscope (BZ-X710, Keyence, Osaka, Japan). The assay was performed according to the manufacturer’s instructions.

### 4.12. miRNA Profiling

CT26 cells were treated with LA (50 μg/mL) for 48 h. Total RNA was extracted using TRI Reagent (Molecular Research Center) according to the manufacturer’s instructions. For miRNA profiling, RNA was sent to the miRXplore microarray service provided by Miltenyi Biotech (Bergisch Gladbach, Germany).

### 4.13. Patients

We obtained frozen tissue samples from 11 patients with CRC with serosal invasion (pT3) and 1–3 regional lymph node metastases (pN1), which were diagnosed at the Department of Molecular Pathology, Nara Medical University, from 2012–2019. Four cases had no distant metastases, three cases showed synchronous liver metastasis, and four cases showed recurrence by liver metastasis more than 2 years after resection of the primary tumors (delayed liver metastasis). As written informed consent was not obtained from the patients for their participation in the present study, all identifying information was removed from patient samples prior to their analysis to ensure strict privacy protection (unlinkable anonymization). All procedures were performed in accordance with the Ethical Guidelines for Human Genome/Gene Research enacted by the Japanese Government and with the approval of the Ethics Committee of Nara Medical University (approval number: 937, 20 October 2014).

### 4.14. Statistical Analysis

Statistical significance was calculated using a two-tailed Fisher’s exact test and ordinary ANOVA using the InStat software (GraphPad, Los Angeles, CA, USA). A two-sided *p*-value of <0.05 was considered to indicate statistical significance.

## Figures and Tables

**Table 1:** Altered microRNA expression in linoleic acid-treated CT26 cells.

miRNA Name	Ratio (Control vs. Linoleic Acid Treatment)
Upregulated	
MIR-7	5.573878
MIR-138-2	2.696476
MIR-21	2.252155
MCMV-MIR-M23-1-5P	2.04248
MIR-1902	2.002737
MIR-494	1.96935
MIR-188-3P	1.793792
MIR-1949	1.667342
MIR-493	1.656996
MIR-720	1.607656
Downregulated	
MIR-210	0.574341

**Table 2:** Primer pairs used in this study.

Gene Symbol	Reference	Forward Primer (5′–3′)	Reverse Primer (5′–3′)
*Mouse miR-494*	Qi et al. [37]	TGGTGATGGGATTTGAAACATACACGGGAAAC	AGATAGACGGTGTCGCTGTTGAAGTCAG
*Mouse S6*	Qi et al. [37]	CTCGCTTCGGCAGCACA	AACGCTTCACGAATTTGCGT
*Mouse Actb*	NM_007393.5 *	ATGTGCCACTCTGACTGGAA	TCCATCGGTCATGCTCTCTC
*Mouse Pgc1α*	BC066868.1 *	ATGTGTCGCCTTCTTGCTCT	ATCTACTGCCTGGGGACCTT
*Mouse MycC*	AH005318.2 *	GCCCAGTGAGGATATCTGGA	ATCGCAGATGAAGCTCTGGT
*Human ACTB*	NM_001101.3 *	GGACTTCGAGCAAGAGATGG	AGCACTGTGTTGGCGTACAG
*Human PGC1α*	BC156323.1 *	GTGAAGACCAGCCTCTTTGC	AATCCGTCTTCATCCACAGG
*Human MYCC*	NM_002467.4 *	TTCGGGTAGTGGAAAACCAG	CAGCAGCTCGAATTTCTTCC

* Gene bank ID.

## Data Availability

Not applicable.

## Abbreviations

LA	linoleic acid
CRC	colorectal cancer
PGC-1α	proliferator-activated receptor γ coactivator 1α
OCR	oxygen consumption rate
ECAR	extracellular acidification rate
ROS	reactive oxygen species
PGE2	prostaglandin E2
NAC	N-acetyl-L-cysteine
